# Current News

**Published:** 1891-07

**Authors:** 


### Current News.

### THE AMERICAN DENTAL SOCIETY OF EUROPE.

THE American Dental Society of Europe will hold its Seventeenth Annual Meeting at Heidelberg-on-the-Neckar, in the Schloss Hotel, August 3, 4, and 5, 1891. The officers for the year are:

Dr. William R. Patton, Cologne, President; Dr. Isaac B. Davenport, Paris, Vice-President; Dr. Lyman C. Bryan, Basel, Secretary; Dr. Charles H. Adams, Frankfort-on-the-Main, Treasurer.

*Executive Committee.*—Drs. Patton, Adams, and Wetzel.

*Membership Committee.*—Drs. Davenport, Jenkins, and Miller.

Members of the profession are cordially invited, and are requested to notify the Secretary at an early date of their intention to attend the meeting, contribute papers, or demonstrate before the Society.

The charming site of Heidelberg will allow the society to intersperse its three days proceedings with excursions to interesting points, and visiting the University and the ruins of the Castle.

---

### NEW JERSEY EXAMINATIONS.

THE New Jersey Dental Commission will hold its next meeting for examinations in Asbury Park, N. J., on Monday, July 13, at 10 A.M.

Persons intending to begin the practice of dentistry in New

Jersey must make application to the Secretary of the Board prior to June 30, 1891.

G. Carleton Brown,
*Secretary.*

Elizabeth, N. J., May 4, 1891.

---

### AMERICAN DENTAL ASSOCIATION.

A resolution was adopted at the last meeting of the American Dental Association, authorizing the Executive Committee to communicate with members of dental societies and with the dental profession of the country, to the end that the membership of the various local and State societies may be increased; that the usefulness of the American Dental Association may be advanced, and the relations between it and the local societies be made more intimate. One object is to awaken a lively interest in the meetings of the American Dental Association, and to secure further representation from the various local societies at the annual general meetings. Will you be kind enough to show your pride in your local society by interesting yourself personally in this matter, and secure the appointment of delegates to the next meeting of the American Dental Association, to be held at Saratoga Springs, N. Y., August 4, 1891? In addition to the appointment of delegates, we would suggest that your society appoint a committee who will forward to the chairman of each section such matters of interest as have transpired in your society during the year. The facts need only to be given to the chairman or secretary in such shape as to enable him to include them in the general report of each section. The names and addresses of the officers of the sections are herewith enclosed. If the societies of which you are a member have already met, will you send the paper you have read, or an abstract of it, or a brief outline of what you said in the course of discussion, or a short report of the entire meeting, to some one of the chairmen or secretaries?

We desire a full attendance at the next meeting, as matters of great importance will come before it. Arrangements will be made to secure reduced railroad rates, but in order to get any reduction for return trip it is *absolutely necessary to get a "certificate plan receipt" for full fare paid* at time of starting, of the agent of whom ticket is purchased. The best hotel rates will be secured, and all who visit Saratoga will, we hope, be well repaid for the time and money spent in visiting that famous resort.

J. N. Crouse,
*Chairman Executive Committee.*

### NATIONAL ASSOCIATION OF DENTAL FACULTIES.

The Eighth Annual Meeting of the National Association of Dental Faculties will be held at Saratoga Springs, New York, on Saturday, August 1, 1891, at 10 o'clock A.M.

Applications for membership must be in the hands of the Executive Committee, Dr. J. Taft, chairman, sixty days prior to the meeting.

Each delegate must be a member of the teaching Faculty in the college he represents, and bring a certificate signed by the president (or dean) and secretary of his college, stating that he is authorized to act for them.

Delegates must be in attendance promptly at 10 A.M. on the day of meeting, in order that all the business may be concluded before the meeting of the American Dental Association.

J. E. Patterson,
*Secretary.*

Keith & Perry Building, Kansas City, Mo.

---

### SOUTHERN DENTAL ASSOCIATION.

The Twenty-third Annual Meeting of the Southern Dental Association will be held at Morehead City, N. C., commencing August 11, 1891. The officers of the association are as follows:

President, Dr. G. F. S. Wright, Georgetown, S. C.; First Vice-President, Dr. R. K. Luckie, Holly Springs, Miss.; Second Vice-President, Dr. W. H. Richards, Knoxville, Tenn.; Third Vice-President, Dr. Louis P. Dotterer, Charleston, S. C.; Recording Secretary, R. C. Marshall, Little Rock, Ark.; Corresponding Secretary, Dr. D. R. Stubblefield, Nashville, Tenn.; Treasurer, Dr. H. E. Beach, Clarksville, Tenn.; Chairman Executive Committee, Dr. H. J. McKellops, St. Louis, Mo.

M. C. Marshall,
*Recording Secretary.*

---

### CONNECTICUT STATE DENTAL ASSOCIATION.

The Twenty-seventh Annual Meeting of the Connecticut State Dental Association was held in Hartford, May 19, at which the following officers were elected: President, Dr. E. S. Gaylord, of New Haven; Vice-President, Dr. C. C. Barker, of Meriden; Secretary, Dr. George L. Parmele, of Hartford; Treasurer, Dr. Joseph H. Smith, of New Haven.

*Executive Committee.*—Dr. James McManus, of Hartford; Dr. William J. Rider, of Danbury; Dr. Daniel A. Jones, of New Haven.

Resolutions of respect to the memory of Dr. William H. Atkinson were read and adopted and a copy ordered to be sent to the family.

George L. Parmele,
*Secretary.*

---

### ALUMNI ASSOCIATION OF THE PHILADELPHIA DENTAL COLLEGE.

At a meeting held April 9, 1891, all the graduates of the Philadelphia Dental College during the years 1886, 1887, 1888, 1889, 1890, and 1891 were elected members. Those desiring to accept such membership will please send to J. R. C. Ward, D.D.S., Treasurer, 1905 Fairmount Avenue, Philadelphia, their name, address, and one dollar entrance fee.

Alonzo Boice, *President.*
L. Ashley Faught, *Secretary.*

---

### ALUMNI ASSOCIATION OF THE NEW YORK COLLEGE OF DENTISTRY.

Officers: President, J. Howard Reed, D.D.S., M.D.S., 1881, 32 West Nineteenth Street, New York City; First Vice-President, John I. Hart, D.D.S., 1886; Second Vice-President, J. W. Taylor, D.D., 1884; Secretary, Vincent M. Munier, D.D.S., 1888, 102 West Ninety-fifth Street, New York City; Treasurer, Zachary T. Sailer, D.D.S., 1880, 40 West Thirty-third Street, New York City.

*Executive Committee.*—Chairman, Sherman B. Price, D.D.S., 1880, 13 West Thirty-second Street, New York City; H. J. Parker, D.D.S., 1883; E. S. Robinson, D.D.S., 1889.

*College Committee.*—Chairman, F. A. Chicherio, D.D.S., 1888, 2852 Atlantic Avenue, Brooklyn, N. Y.; George A. Hull, D.D.S., 1888; Edmund D. Frost, D.D.S., 1886; Arthur L. Swift, D.D.S., 1885; J. J. Strohmeyer, D.D.S., 1884.

---

### ILLINOIS STATE DENTAL SOCIETY.

At the Twenty-seventh Annual Meeting of the Illinois State Dental Society, held at Bloomington, May 12 to 15, 1891, the following officers were elected for the ensuing year: President, W. H. Taggart, Freeport; Vice-President, Garret Newkirk, Chicago; Secretary, Louis Ottofy, Chicago; Treasurer, W. A. Stevens, Chicago; Librarian, F. H. McIntosh, Bloomington. The next meeting will be held in Springfield, beginning on the second Tuesday in May, 1892.

LOUIS OTTOFY,
*Secretary.*

### LIST OF HOUSES AT SARATOGA SPRINGS, N. Y.

THE following places at Saratoga are recommended. The terms of all are reasonable and in many respects they are more comfortable than the hotels.

| Name. | Proprietor. | Day. | Week. | Address. | Distance from Hall. |
|---|---|---|---|---|---|
| Temple Grove . . | A. B. Dowd. | $3.00. | $12.00–$20.00. | Saratoga Springs. | 5 blocks. |
| Huestis House . | W. B. Huestis. | $3.00. | $17.50. | South Broadway, Saratoga Sp'gs. | 5 " |
| Kenmore . . . . | J. N. Ramsdill. | $2.00. | | Saratoga Springs, N. Y. | 2 " |
| Albemarle* . . . | W. J. Riggs. | | | | 5 " |
| Balch House . . | W. S. Balch. | | $12.50–$17.50. | 526 North Broadway, Saratoga Springs. | 1½ " |
| Broadway House | S. Hine. | $2.00. | $10.00–$14.00. | 522 Broadway, Saratoga Springs. | 1 " |
| Howland House . | J. Howland. | $2.00–$2.50. | $10.00–$15.00. | 573 North Broadway, Saratoga Springs. | 3 " |
| Foley House . . | Miss M. C. Foley. | $2.50. | $14.00. | 226 South Broadway, Saratoga Springs. | 5 " |
| Garden View . . | Mrs. T. S. Carpenter. | | | | 1 " |
| Lafayette House . | Mrs. M. A. Root. | $2.00–$2.50. | $10.00–$15.00. | Corner of Lafayette and Circular. | 4 " |
| The Linwood . . | S. M. Van Deusen. | $2.00–$2.50. | $14.00–$17.50. | South Broadway, Saratoga. | 5 " |
| Trim Cottage . . | Mrs. H. P. Trim. | | | | 4 " |
| Tefft House . . . | T. T. Tefft. | | | | 4 " |
| Summer Rest . . | Mr. M. E. Morse. | $2.00–$2.50. | $10.00–$15.00. | Saratoga Springs, N. Y. | 5 " |
| Vermont House . | Mrs. C. M. Dyer. | | | | 1½ " |
| Washington Hall | Mr. A. J. Starr. | $2.50. | $12.00–$15.00. | 587 North Broadway, Saratoga. | 3 " |

* Those left blank have not responded as to terms. The rate per week is for one person.—[ED.]

### AMERICAN DENTAL ASSOCIATION.

THE Thirty-first Annual Session of the American Dental Association, will be held at Saratoga Springs, N. Y., commencing Tuesday, August 4, at 10 o'clock A.M.

GEORGE H. CUSHING,
*Secretary.*

### COLLEGE COMMENCEMENT, UNIVERSITY OF PENNSYLVANIA, DEPARTMENT OF DENTISTRY.

The commencement of the Departments of Medicine and Dentistry took place at the Academy of Music, Philadelphia, May 1, 1891. The degrees were conferred by the provost, Professor William Pepper. The address tc the graduates was given by Professor James Tyson. Number of matriculates, 206; absent, 2; deceased, 1; graduates, 80.

### UNION MEETING OF THE NEW JERSEY AND PENNSYLVANIA STATE DENTAL SOCIETIES.

The joint union meeting of the New Jersey and Pennsylvania State Dental Societies will convene at the West End and Ocean Hotels, Asbury Park, N. J., Wednesday evening, July 15, at eight o'clock, and continue in session Thursday and Friday. No society business will be transacted at this meeting, and the most eminent men in the profession will read interesting papers. The clinics will be the most varied and newest in operative and mechanical dentistry. Two large halls, connected, have been provided for meetings, clinics, and exhibits. The Twenty-third Annual Meeting of the Pennsylvania State Society for the transaction of general business will convene at Justi's Dental Hall, Philadelphia, 10 A.M., Tuesday, July 14. The Twenty-first Annual Meeting and anniversary of the New Jersey State Dental Society will meet at the above-named hotels, Asbury Park, Wednesday, July 15, 10 A.M., for general business, the afternoon devoted to anniversary exercises. Full programmes will be mailed on application to Dr. Frank L. Bassett, Girard building, Philadelphia, or the secretaries.

C. V. Kratzer, D.D.S.,
*Secretary for Pennsylvania, Reading.*
Charles A. Meeker, D.D.S.,
*Secretary for New Jersey, Newark.*

### RAILROAD FARES TO THE AMERICAN DENTAL ASSOCIATION.

Dr. Crouse writes at the last moment, before going to press, that he has not yet completed railroad arrangements, but says, "Am quite sure that I shall be able to secure the *one and a third fare on most lines.*"

It is of importance for those paying full fare *to take receipt. This will entitle the party to whatever reduction Dr. Crouse may be able to secure.*

